# Beyond Fingersticks: Continuous Glucose Monitoring for Inpatient Glycemic Management in a Patient With Type 1 Diabetes and Castleman Disease—A Case Report

**DOI:** 10.1155/crie/1901927

**Published:** 2026-11-14

**Authors:** Cristian D. Armas, Amna Kamran, Manasi Shah

**Affiliations:** ^1^ Division of Endocrine and Metabolic Disorders Department of Medicine Macon and Joan Brock Virginia Health Sciences at Old Dominion University Norfolk Virginia USA odu.edu

**Keywords:** care report, Castleman disease, continuous glucose monitoring, type 1 diabetes

## Abstract

Continuous glucose monitoring (CGM) provides real‐time interstitial glucose data and may detect glycemic excursions missed by point‐of‐care blood glucose (POC BG) testing. Inpatient evidence remains limited, especially in patients with severe dermatologic disease limiting POC access. We report a 22‐year‐old male with poorly controlled type 1 diabetes and Castleman disease who presented with diffuse pruritic hyperpigmented lesions, mucositis, and nail dystrophy, precluding reliable fingerstick monitoring. During his several‐month hospitalization, he received high‐dose corticosteroids, rituximab, and intravenous immunoglobulin, with marked glycemic variability due to steroid‐induced hyperglycemia, variable intake, and intermittent refusal of care. A Dexcom G7 CGM was initiated to facilitate glucose monitoring, and a single earlobe capillary glucose measurement was obtained at the time of CGM initiation as a reference blood glucose value. CGM data were manually integrated into the electronic medical record (EMR) by nursing staff and used to support insulin titration. Most serum and POC BG measurements did not show major differences from corresponding CGM values. During the first month, time in range (TIR) was 30%, time below range was 2%, and glucose management indicator (GMI) was 8.9%. CGM was used for nearly 3 months during hospitalization, facilitating CGM when POC testing was challenging. The patient later developed respiratory failure, likely due to bronchiolitis obliterans, and died. CGM enabled continuous monitoring when fingersticks were not feasible, supporting insulin adjustment in complex inpatient hyperglycemia. Implementation requires multidisciplinary coordination and validation protocols.

## 1. Introduction

Continuous glucose monitoring (CGM) uses a subcutaneous sensor to provide real‐time interstitial glucose readings, unlike intermittent point‐of‐care blood glucose (POC BG) testing [1, 2]. It is the standard of care in outpatient type 1 diabetes mellitus (T1DM) and is increasingly used in type 2 diabetes [3].

CGM accuracy may be affected by perfusion, hydration status, temperature, and rapid glycemic shifts, limiting its reliability in acutely ill patients [4]. It is generally not recommended in critically ill or septic patients, and dermatologic conditions such as active skin infections may also interfere with sensor performance [4, 5]. Limited inpatient experience and robust data have restricted U.S. FDA approval and widespread hospital use. Only a few randomized controlled trials (RCTs) have evaluated inpatient CGM, including during the COVID‐19 pandemic, when the FDA temporarily allowed its use to reduce exposure and conserve resources [6].

We report a case of a patient with T1DM and Castleman disease whose extensive dermatologic lesions made reliable fingerstick POC BG testing challenging. This case provides a unique clinical context for inpatient CGM use, illustrating its potential to facilitate CGM when conventional fingerstick testing is difficult to perform.

## 2. Case Report

A 22‐year‐old male with poorly controlled T1DM (A1c 12.7%) presented with a 6‐month history of diffuse hyperpigmented, pruritic skin lesions involving the face, trunk, extremities, palms, fingers, and genitalia, accompanied by erosive oral and ocular mucositis and nail dystrophy (Figure 1). Skin biopsy with direct immunofluorescence was consistent with paraneoplastic pemphigus. CT imaging revealed a pelvic mass, which was surgically resected with regional lymph nodes; pathology confirmed Castleman disease.

**Figure 1:**
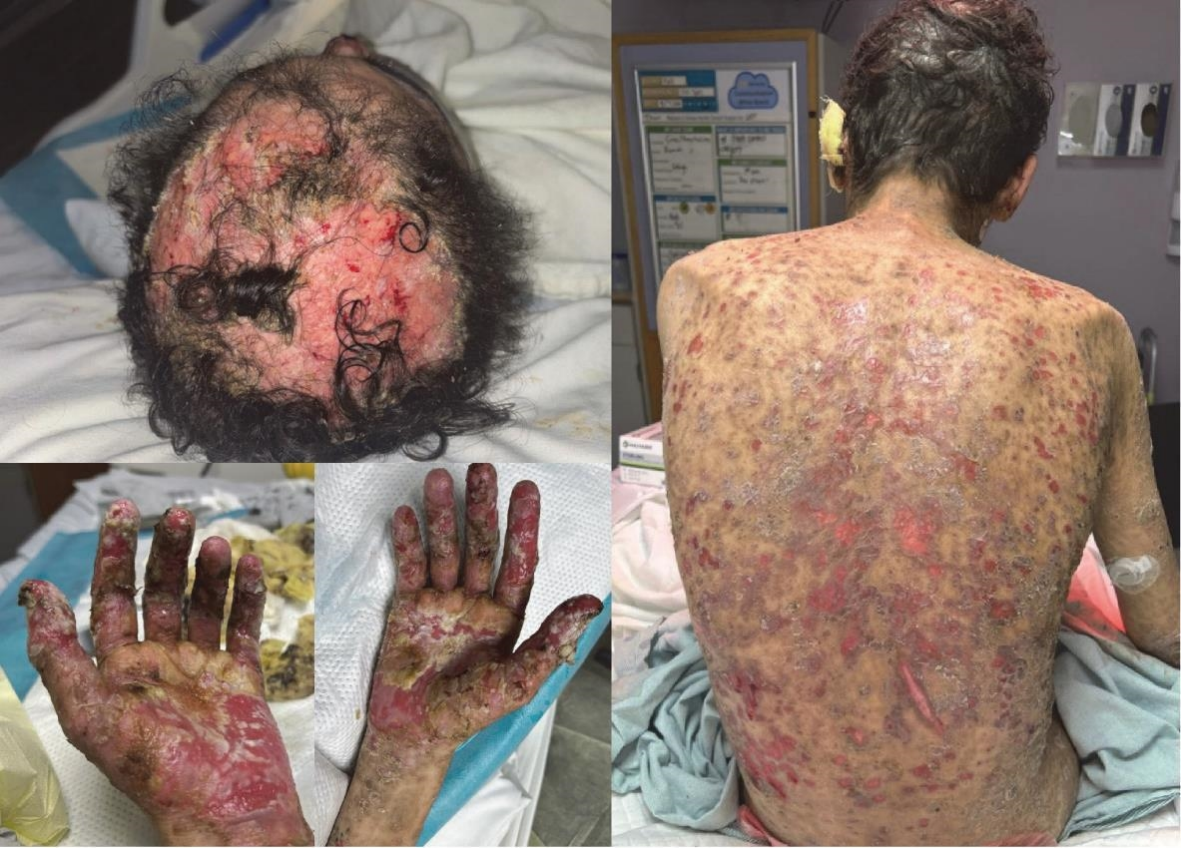
Paraneoplastic pemphigus with extensive skin lesions associated with Castleman disease, including severe polymorphous cutaneous findings such as lichenoid lesions, blisters, and generalized violaceous papules and macules.

During hospitalization, he was treated with high‐dose systemic corticosteroids (IV methylprednisolone 40–50 mg daily, later converted to prednisone, with a 20 mg taper at discharge), tocilizumab, rituximab, and IVIG. Glycemic management was complicated by glucocorticoid‐induced hyperglycemia and difficulty obtaining fingerstick POC BG measurements due to severe digital involvement. Glucose levels were highly labile, ranging from the 70s to the 400s mg/dL despite multiple insulin adjustments (Figure 2).

**Figure 2:**
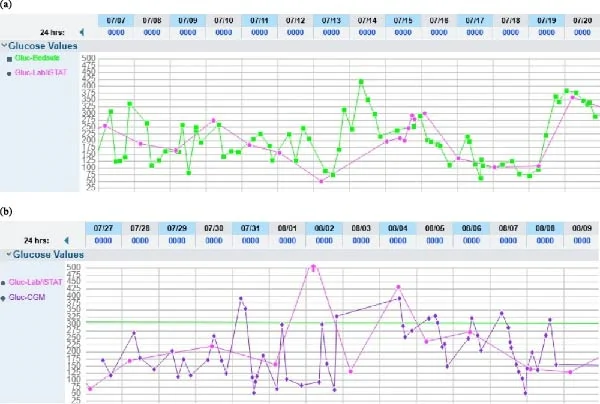
Glucose patterns during the 14 days before (a) and after (b) CGM initiation. Marked glycemic variability was observed throughout this period. Many of the available serum glucose measurements showed no major differences from the corresponding CGM glucose values.

A Dexcom G7 CGM was initiated to minimize skin trauma and facilitate CGM (Figure 3). A single earlobe POC BG measurement was obtained at the time of CGM initiation as a reference blood glucose value. CGM data were manually integrated into the electronic medical record (EMR) Epic by the nursing staff. Available CGM readings were generally similar to the corresponding POC BG and laboratory plasma glucose measurements (Figure 2).

**Figure 3:**
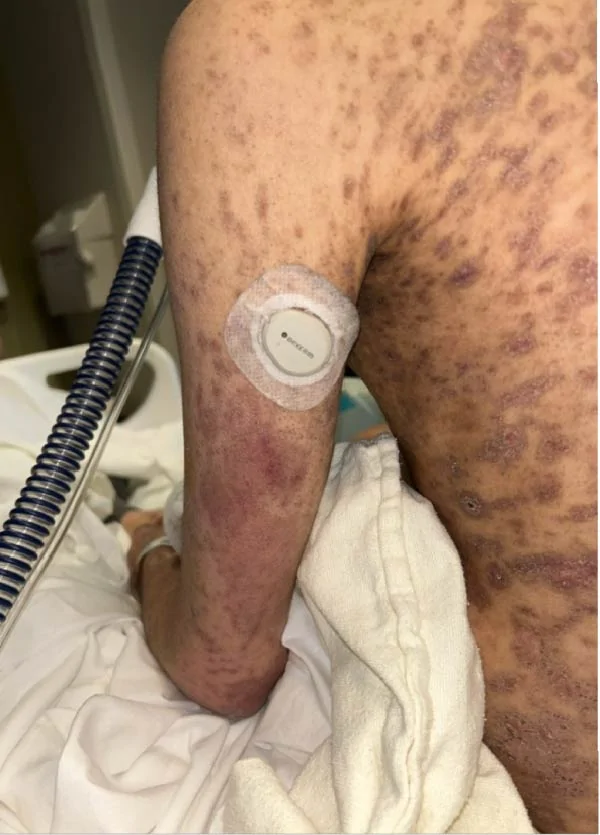
A Dexcom G7 CGM was placed on the posterior aspect of the left arm.

Glycemic control remained challenging due to fluctuating corticosteroid dosing, intermittent patient refusal of steroids, and variable dietary patterns. CGM data facilitated daily insulin titration, including adjustments to basal insulin, insulin‐to‐carbohydrate ratios, and insulin sensitivity factors, particularly in response to nocturnal glycemic variability. His basal insulin requirements fluctuated substantially, ranging from 20 to 15 units and occasionally requiring a reduction to as low as 3 units. During the first 14 days of CGM use, average glucose was 187 mg/dL, glucose management indicator (GMI) was 7.8%, time in range (TIR) (70–180 mg/dL) was 53%, with 22% of readings in the high range (181–250 mg/dL), 22% in the very high range (>250 mg/dL), 2% in the low range (54–69 mg/dL), and 1% in the very low range (<54 mg/dL) (Figure 2). After 1 month of CGM use, GMI was 8.9%, with 30% TIR and 2% time below the range (TBR) (<70 mg/dL). CGM was continued for nearly 3 months during the hospitalization, supporting CGM when POC testing remained challenging. No diabetic ketoacidosis was seen. The patient was discharged; however, 6 days later, he was readmitted with complicated respiratory failure, likely secondary to bronchiolitis obliterans. He was intubated in the ICU and subsequently passed away.

## 3. Discussion

Over the past decade, minimally invasive glucose monitoring technologies have markedly transformed diabetes care through the use of CGM devices [7]. In hospitalized patients, CGM algorithms can predict glycemic excursions and enable earlier intervention—particularly for nocturnal hypoglycemia—when combined with confirmatory POC testing [8]. Despite these advantages, POC BG remains the gold standard [9]. However, blinded CGM studies show that POC testing may miss up to 33% of hyperglycemic events and up to 90% of hypoglycemic episodes [10, 11]. CGM can detect hypoglycemia 3–4 h earlier, including severe nocturnal events, and may identify adverse glycemic events that are missed by POC testing, with approximately one‐quarter of CGM‐detected events not being identified by POC testing alone [12, 13].

In this case, inpatient glycemic management was challenging due to severe digital skin involvement from Castleman disease, which limited fingerstick POC testing. A Dexcom G7 CGM was initiated, with glucose values showing no major differences from the available daily fasting serum chemistry and intermittent earlobe POC measurements. Evidence on CGM use in patients with pre‐existing skin disease is limited. A prospective study of patients with pemphigus, bullous pemphigoid, or severe eczema receiving high‐dose glucocorticoids found that CGM detected steroid‐induced hyperglycemia in 47.1% of cases, including patients who were initially normoglycemic. These findings suggested that CGM may help identify previously unrecognized hyperglycemia in patients with severe skin disease receiving glucocorticoids [14]. In our patient, CGM continued to provide glucose readings despite changes in the extent of the patient’s skin lesions. However, because this was a single case without a formal assessment of CGM accuracy, the reliability of these findings cannot be established. Factors such as sensor placement, lesion distribution, and changes in the skin condition may have affected the CGM performance.

RCTs have also evaluated the potential benefits of inpatient CGM. In a trial of 110 non‐ICU patients with T2DM, CGM significantly improved glycemic control compared with POC testing, with a lower mean glucose (218.5 mg/dL), 11.41% less time spent in severe hyperglycemia (>250 mg/dL), and increased time within the target range (70–250 mg/dL) [15]. Another RCT combining CGM with a glucose telemetry system that wirelessly transmits values to a centralized nursing monitor demonstrated significant reductions in both overall and clinically significant hypoglycemia [16]. Together, these studies suggest that CGM may provide additional information beyond intermittent POC BG measurements.

CGM accuracy is an important consideration for clinical decision‐making in hospitalized patients. Prior studies of Dexcom G6 report a strong correlation with POC BG (*R* = 0.90–0.95) and a mean absolute relative difference of approximately 9%–10% [17, 18]. A retrospective study comparing CGM with POC BG in patients receiving basal‐bolus or IV insulin found CGM‐based correction dosing to be clinically comparable to POC testing. Although CGM values were consistently lower than POC (mean difference 44.8 mg/dL for basal bolus and 19.7 mg/dL for IV insulin), overall agreement was high (83%) [12]. Recent multicenter data provide additional support for inpatient CGM use in patients with T1DM. Wang et al. [19] evaluated CGM accuracy in hospitalized adults with T1DM and found that CGM provided clinically relevant glucose measurements in the inpatient setting. In our patient, most laboratory and POC BG measurements did not appear markedly different from the CGM values; however, no formal accuracy analysis was performed.

Optimal inpatient glycemic targets are 140–180 mg/dL, and achieving tighter control remains challenging, even with CGM. In a recent RCT of noncritically ill adults with T2DM, CGM‐guided intensive glycemic targets did not significantly improve mean glucose compared with standard care, and few patients achieved tighter goals [20]. In our case, the TIR was 30% during the first month of CGM use. However, the clinical applicability of TIR in hospitalized patients is limited as glycemia fluctuates rapidly with acute illness and treatment changes [4]. Accordingly, avoidance of hypoglycemia (<70 mg/dL) remains the primary goal, with prevention of severe hyperglycemia (<54 mg/dL) as a secondary objective. In our patient, 2% of readings were below 70 mg/dL during the first month of CGM use, including 1% below 54 mg/dL during the first 14 days, while no DKA occurred.

Beyond conventional CGM metrics, additional approaches to interpreting glucose data may provide further insight into inpatient glycemic patterns. CGM‐derived dynamic markers may capture aspects of glucose regulation not reflected by measures such as mean glucose or TIR [21]. Machine learning models may similarly provide new approaches for identifying clinically relevant patterns from CGM and other patient‐level data [22]. These approaches may represent future avenues for improving inpatient glycemic assessment and supporting more individualized care.

Finally, successful inpatient CGM implementation requires coordinated involvement of a multidisciplinary team and clear protocols for documentation and POC BG corroboration. CGM values should be verified with POC testing in situations of extreme glucose values, hypoglycemic symptoms, hemodynamic instability, or perioperative care [23]. As most CGM systems are not yet integrated into hospital EMRs, institutions must establish standardized workflows. In our case, CGM data were manually documented in the EMR (Epic), and nursing staff were trained on appropriate POC confirmation when clinically indicated.

## 4. Conclusions

Current evidence supports the potential role of CGM in inpatient glucose monitoring, particularly when conventional POC testing is challenging. In this case, CGM provided continuous glucose data despite extensive skin disease that limited fingerstick POC testing. However, the findings are based on a single case without a formal assessment of CGM accuracy and should therefore be interpreted cautiously. Further studies are needed to evaluate the accuracy, clinical utility, and appropriate metrics for inpatient CGM use, as well as to establish standardized workflows for EMR integration and staff training.

## Author Contributions

Manasi Shah and Amna Kamran were involved in the clinical management of the patient, contributed to data collection and follow‐up, and assisted with the literature review and manuscript editing. Cristian D. Armas clinically reviewed the patient, contributed to the conception and design of the case report, and wrote the first draft of the manuscript.

## Funding

No funding was received for this manuscript.

## Disclosure

All authors have read and approved the final version of the manuscript. Cristian D. Armas had full access to all of the data in this study and takes complete responsibility for the integrity of the data and the accuracy of the data analysis.

## Consent

Written informed consent was obtained from the patient’s mother for the publication of this case report.

## Conflicts of Interest

The authors declare no conflicts of interest.

## Data Availability

The data supporting the findings of this study are available from the corresponding author upon reasonable request. The data are not publicly available due to ethical and privacy considerations.
